# A novel nomogram for predicting the recurrence of atrial fibrillation in patients treated with first-time radiofrequency catheter ablation for atrial fibrillation

**DOI:** 10.3389/fcvm.2024.1397287

**Published:** 2024-08-21

**Authors:** Guiling Ma, Changhong Zou, Zhiyong Zhang, Lin Zhang, Jianjun Zhang

**Affiliations:** ^1^ Department of Cardiology, Beijing Chaoyang Hospital, Capital Medical University, Beijing, China; ^2^ Heart Center and Beijing Key Laboratory of Hypertension, Beijing Chaoyang Hospital, Capital Medical University, Beijing, China

**Keywords:** atrial fibrillation, radiofrequency catheter ablation, recurrence, anti-M2-R, prediction model

## Abstract

**Introduction:**

The purpose of this study was to investigate the predictive factors of atrial fibrillation (AF) recurrence in patients after first-time radiofrequency catheter ablation (RFCA) and to develop a nomogram predictive model that can provide valuable information for determining the ablation strategy.

**Methods:**

In total, 500 patients who had received first-time RFCA for AF were retrospectively enrolled in the study. The patients were divided into a training cohort (*n* = 300) and a validation cohort (*n* = 200) randomly at a 6:4 ratio. Lasso and multivariate logistic regression analyses were used to screen the predictors for AF recurrence during a 2-year follow-up. The C-index and a calibration plot were used to detect the discriminative ability and calibration of the nomogram. The performance of the nomogram was assessed compared with the APPLE score, CAAP-AF score, and MB-LATER score using the receiver operating characteristic (ROC) curve, decision curve analysis (DCA), integrated discrimination index (IDI), and net reclassification index (NRI).

**Results:**

A total of 78 patients experienced the recurrence of AF after first-time RFCA in the training cohort. The six strongest predictors for AF recurrence in the training cohort were persistent AF, duration of AF, left atrial diameter (LAD), estimated glomerular filtration rate (eGFR), N-terminal pro-brain natriuretic peptide (NT-proBNP), and autoantibody against M2-muscarinic receptor (anti-M2-R). Based on the above six variables, a nomogram prediction model was constructed with a C-index of 0.862 (95% CI, 0.815–0.909), while the C-index was 0.831 (95% CI, 0.771–0.890) in the validation cohort. DCA showed that this nomogram had greater net benefits compared with other models. Furthermore, the nomogram showed a noticeable improvement in predictive performance, sensitivity, and reclassification for AF recurrence compared with the APPLE score, CAAP-AF score, or MB-LATER score.

**Conclusion:**

We established a novel predictive tool for AF recurrence after the first-time RFCA during a 2-year follow-up period that could accurately predict individual AF recurrence.

## 1 Introduction

Atrial fibrillation (AF) is one of the most common arrhythmia in clinical practice, and it is associated with an increased risk of death, stroke, and embolism. The prevalence of AF ranges from 2% in the general population to 10%–12% in individuals aged 80 years or more (1). The estimated population of AF reached 50 million individuals in 2020 (2). Radiofrequency catheter ablation (RFCA) has become one of the most effective treatment strategies for restoring sinus rhythm in AF patients (3, 4). With the improvement of techniques and technologies of RFCA, previous studies have shown that the recurrence of AF after first-time RFCA varies from 11% to 29% in paroxysmal AF and up to 70% in persistent AF during a 5-year follow-up period (5, 6). Hence, it is important to confirm predictors for the recurrence of AF, as this information would be helpful in increasing the success rate of RFCA and providing guidelines for clinical practice.

Several studies have considered the factors that affect atrial remodeling and are involved in the pathogenesis of AF as an index to predict the AF recurrence, including APPLE score, CAAP-AF score, and MB-LATER score (7–9). However, the predictive value of these factors and scores for the recurrence of AF after RFCA is still controversial. Autoantibodies against M2-muscarinic receptor (anti-M2-R) are closely related to the occurrence and development of AF (10–13), and we found that anti-M2-R was one of the independent factors for the recurrence of AF during the 1-year follow-up (10). However, there have been few relevant studies focusing on the role of anti-M2-R in constructing a nomogram prediction model for the recurrence of AF after first-time RFCA.

In this research, Lasso and multivariate logistic regression analyses were used to screen predictive factors for the recurrence of AF after first-time RFCA during a 2-year follow-up. We constructed a nomogram model to predict the risk of AF recurrence. Besides, we compared the nomogram model with the APPLE score, CAAP-AF score, and MB-LATER score in terms of the predictive performance, sensitivity, and reclassification for AF recurrence.

## 2 Materials and methods

### 2.1 Study population

**Figure 1:**
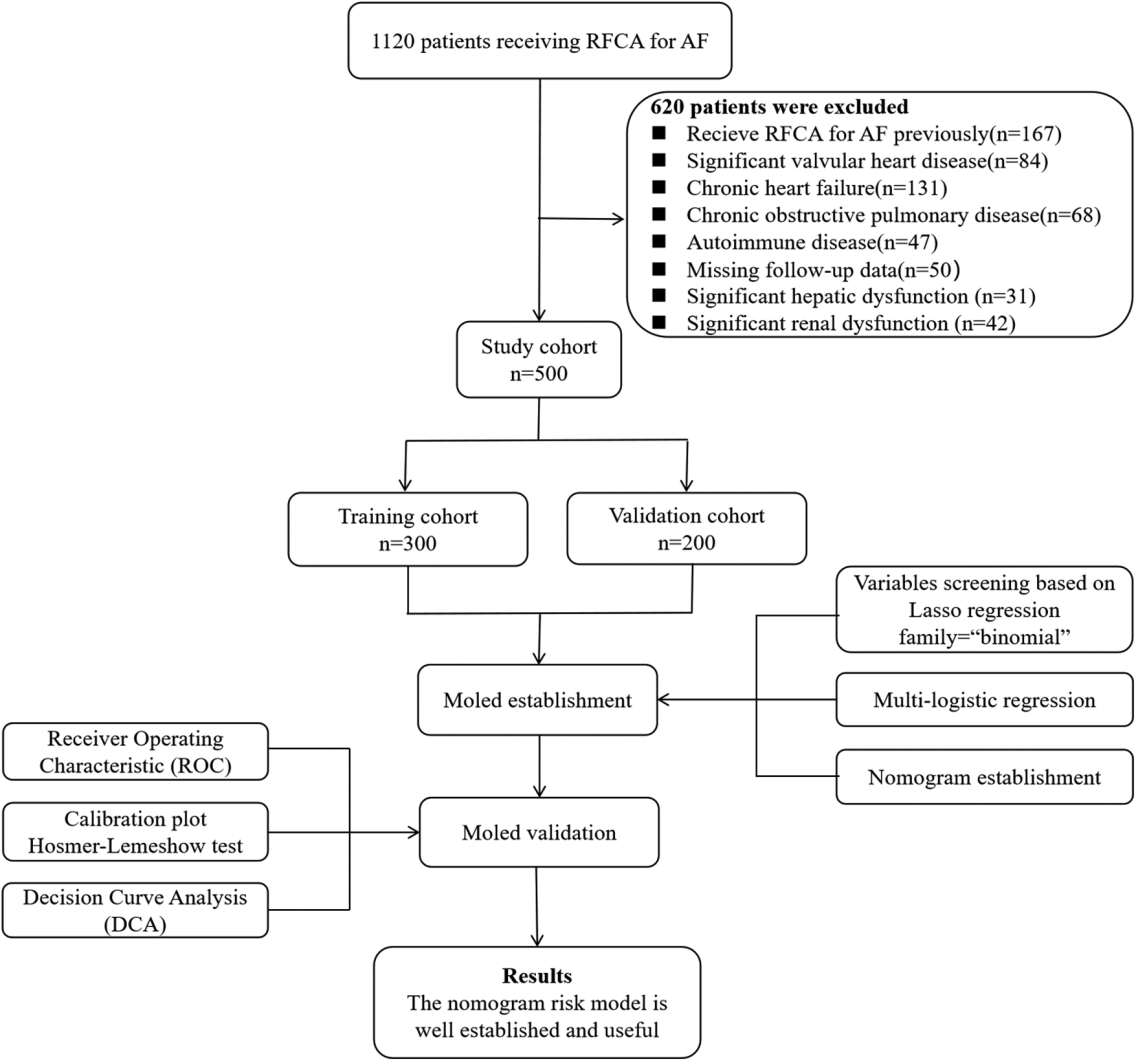
Flowchart of the selection of patients, construction of the model, and validation process.

In this study, the data analyzed were acquired from the Hospital Information System of Beijing Chaoyang Hospital, Capital Medical University, from January 2019 to June 2021. We screened the medical records of 1,120 patients admitted to our medical center for receiving RFCA for AF. The inclusion criteria were as follows: age between 18 and 80 years; paroxysmal or persistent AF; and successfully performed RFCA for AF. The exclusion criteria were as follows: previous RFCA for AF; significant valvular heart disease; chronic heart failure; chronic obstructive pulmonary disease; autoimmune disease, including hyperthyroidism; missing follow-up data; or moderate to severe hepatic or renal dysfunction. We ultimately included 500 patients in this research (Figure 1). The study was conducted in accordance with the Declaration of Helsinki and was approved by the Ethics Committee of Beijing Chaoyang Hospital, Capital Medical University (2023-S-454).

### 2.2 Ablation procedure

Transthoracic echocardiography was performed to record the left-atrial diameter (LAD) in the four-cavity view, and left-ventricular end-diastolic diameter (LVEDD) and left-ventricular ejection fraction (LVEF) in the M mode. Transesophageal echocardiography was arranged to ensure the absence of intracardiac thrombus. Using a three-dimensional electro-anatomical mapping system, CARTO, continuous circumferential ablation that encircled the pulmonary vein ostia was performed. If AF persisted or recurred during paced rhythm after pulmonary vein isolation, additional substrate modification could be required. The endpoint of ablation was considered to be a bi-directional conduction block around linear ablation lesions. Patients were heparinized during the ablation procedure, maintaining an activation coagulation time of 250–350 s. After ablation, patients underwent electrocardiogram telemetry monitoring for 24 h.

### 2.3 Follow-up examination

After discharge, patients were scheduled for regular visits in an outpatient clinic at 3, 6, 12, 18, and 24 months. During each visit, patients underwent physical examination, were intensively questioned about arrhythmia-related symptoms, and underwent a 12-lead electrocardiogram. Meanwhile, a 24-h Holter recording was regularly performed every six months. The recurrence of AF was defined as any symptomatic or detected episode of AF, atrial flutter, or atrial tachycardia longer than 30 s beyond the blanking period of 3 months (14).

### 2.4 Data collection and extraction

Five hundred eligible patients were randomly assigned to a training cohort and a validation cohort at a 6:4 ratio. General data, relevant information about AF, and medical history were extracted from the electronic medical records. Moreover, echocardiography data and laboratory results including hemoglobin, alanine aminotransferase, creatinine, NT-proBNP, and anti-M2-R were collected. The selection of putative predictors was based on previous studies and the investigators' clinical experience.

### 2.5 Model construction and validation

We used 10-fold cross-validation Lasso regression for variable selection among 27 variables. The variables included in the final model was determined based on the location of the corresponding lambda.1SE. Multivariate logistic regression was used to screen the independent factors for AF recurrence among the variables selected above. Then, a nomogram was established. The C-index was used to assess discrimination ability and the calibration curve was used to evaluate the calibration ability of the nomogram. Meanwhile, bootstrap resampling (bootstrap = 500) was used in the internal validation. To evaluate the practicality and clinical utility of the nomogram, we utilized decision curve analysis (DCA). A flowchart illustrating the model construction and validation process is shown in Figure 1.

### 2.6 Multimodel comparison

We also calculated several other classic prediction scores for AF recurrence after RFCA therapy, including the APPLE score [age > 65 years, persistent AF, impaired eGFR (<60 ml/min/1.73 m^2^), LAD ≥ 43 mm, EF < 50%] (7), CAAP-AF score (coronary artery disease, atrial diameter, age, persistent or long-standing AF, number of antiarrhythmic drugs failed, and female sex) (8), and MB-LATER score [male sex, bundle branch block, LAD ≥ 47 mm, type of AF (paroxysmal, persistent, or long-standing persistent), and early recurrent AF] (9). Finally, the integrated discrimination improvement (IDI) and net reclassification improvement (NRI) indices were calculated to compare the performance of our nomogram with the above models.

### 2.7 Statistical analysis

Quantitative data were expressed as the mean ± standard deviation (SD) or median (Q1, Q3). Categorical variables were expressed as frequencies and percentages. Differences between the two groups were compared by the unpaired Student's *t*-test or the Mann-Whitney *U*-test for quantitative data and the chi-square test for categorical variables. Lasso regression was performed using the “glmnet” package for variable shrinkage and selection. The **“**glm” package was used for multivariate logistic regression analysis. The nomogram, DCA, and calibration curve were plotted by R package “rms.” The ROC curve was plotted by the “ggROC” package. The IDI and NRI indices of multiple models were compared separately using the “PredictABEL” and “nricens” packages. Two-tailed *P*-values lower than 0.05 was considered statistically significant. Statistical analysis was performed by R 4.2.1.

## 3 Results

### 3.1 Patient profiles

The clinical characteristics of the 500 patients, including 300 patients in the training cohort and 200 patients in the validation cohort, are presented in Table 1. The median age was 64.2 ± 7.4 years. A total of 337 patients (67.4%) had paroxysmal AF, and 163 patients (32.6%) had persistent AF. The median course of AF was 2.0 years, and the median CHA_2_DS_2_-VASc score was 2. The baseline data were matched between the two cohorts.

**Table 1:** Clinical characteristics of the study population.

Variables	All patients (*n* = 500)	Training cohort (*n* = 300)	Validation cohort (*n* = 200)	*P*-value
Age (years)	64.2 ± 7.4	64.0 ± 7.0	64.5 ± 7.8	0.432
Sex, male (%)	264 (52.8%)	151 (50.3%)	113 (55.6%)	0.207
Persistent AF, *n* (%)	163 (32.6%)	100 (33.3%)	63 (31.5%)	0.741
Course of AF (years)	2.0 (1.0; 3.0)	2.0 (1.0; 3.0)	2.0 (1.0; 3.0)	0.358
Hypertension, *n* (%)	292 (58.4%)	179 (59.7%)	113 (56.5%)	0.541
Diabetes, *n* (%)	132 (26.4%)	79 (26.3%)	53 (26.5%)	0.967
Stroke, *n* (%)	87 (17.4%)	54 (18.0%)	33 (16.5%)	0.754
CAD, *n* (%)	109 (21.8%)	63 (21.0%)	46 (23.0%)	0.674
Smoke, *n* (%)	163 (32.6%)	105 (35.0%)	58 (29.0%)	0.192
Drink, *n* (%)	98 (19.6%)	55 (18.33%)	43 (21.5%)	0.448
BMI (kg/m^2^)	23.83 ± 1.76	23.73 ± 1.84	23.98 ± 1.62	0.125
LAD (mm)	49.78 ± 5.15	49.52 ± 5.45	50.16 ± 4.66	0.166
LVEDD (mm)	47.41 ± 3.46	47.48 ± 3.23	47.30 ± 3.78	0.571
LVEF (%)	65.30 ± 6.52	65.70 ± 6.42	64.71 ± 6.63	0.097
HGB (g/L)	131.87 ± 15.98	131.43 ± 15.02	132.54 ± 17.35	0.448
ALT (U/L)	21.47 ± 8.33	20.94 ± 6.21	20.96 ± 7.29	0.972
eGFR (ml/(min × 1.73 m^2^)	89.47 ± 8.27	89.62 ± 7.31	89.25 ± 9.55	0.635
Anti-M2-R (pmol/L)	109 (94; 153)	106.5 (94; 150)	119 (94; 157)	0.306
NT-proBNP (pg/ml)	151 (121; 183)	155 (129; 181)	145 (117; 187)	0.413
CHA_2_DS_2_-VASc score	2 (1; 3)	2 (1; 3)	2 (1; 3)	0.829

AF, atrial fibrillation; CAD, coronary artery disease; BMI, body mass index; LAD, left-atrial diameter; LVEDD, left-ventricular end-diastolic diameter; LVEF, left-ventricular ejection fraction; HGB, hemoglobin; ALT, alanine aminotransferase; eGFR, estimated glomerular filtration rate; Anti-M2-R, autoantibody against M2-muscarinic receptor; NT-proBNP, N-terminal pro-brain natriuretic peptide.

### 3.2 Variables selection and model construction

**Figure 2:**
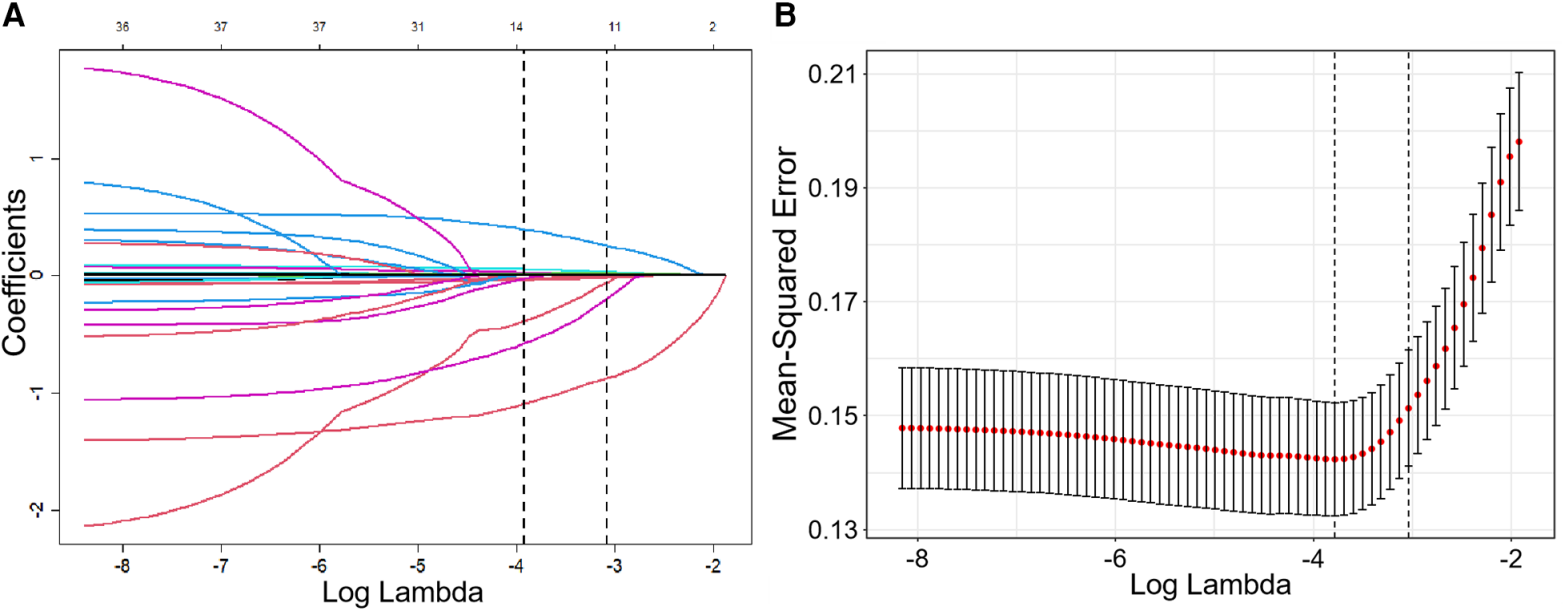
Variable shrinkage and selection using Lasso regression. **(A)** As the value of Log Lambda increases, the prediction error of the model increases simultaneously, while the number of independent variables decreases accordingly. **(B)** Cross-validation Lasso with 10-fold for variables selection. For the optimal parameter, we used lambda. 1SE for variable selection.

The new prediction model was developed in the training cohort with 78 cases of AF recurrence. Lasso regression with 10-fold cross-validation was performed for variable selection. Ten variables, including persistent AF, course of AF, alcohol consumption, coronary artery disease, eGFR, LAD, LVEDD, LVEF, NT-proBNP, and anti-M2-R, were selected based on the lambda.1SE with the criteria of “family = binomial”. The variable shrinkage and cross-validation results are shown in Figure 2. After multivariate logistic regression with the “backward” method, six variables were selected in this model. The regression equation of the model was: L = Persistent AF + Course of AF × 0.463/1.512 + LAD × 0.098/1.512−eGFR × 0.074/1.512 + NT-proBNP × 0.009/1.512 + Anti-M2-R × 0.008/1.512. The risk factors detected by multivariate logistic regression analysis for the recurrence of AF after first-time RFCA are shown in Table 2.

**Table 2:** Multivariate logistic analysis for AF recurrence after catheter ablation.

	B	SE	Wald	*P*-value	OR	95% CI
Persistent AF	1.512	0.336	4.504	<0.001	4.538	2.375–8.907
Course of AF	0.463	0.114	4.051	<0.001	1.588	1.276–2.000
LAD	0.098	0.031	3.184	0.001	1.103	1.039–1.173
eGFR	−0.074	0.024	−3.136	0.002	0.929	0.884–0.971
NT-proBNP	0.009	0.003	3.544	<0.001	1.009	1.004–1.014
Anti-M2-R	0.008	0.002	3.495	<0.001	1.008	1.003–1.013

OR, odds ratio; CI, confidence interval; AF, atrial fibrillation; LAD, left-atrial diameter; eGFR, estimated glomerular filtration rate; NT-proBNP, N-terminal pro-brain natriuretic peptide; Anti-M2-R, autoantibody against M2-muscarinic receptor.

**Figure 3:**
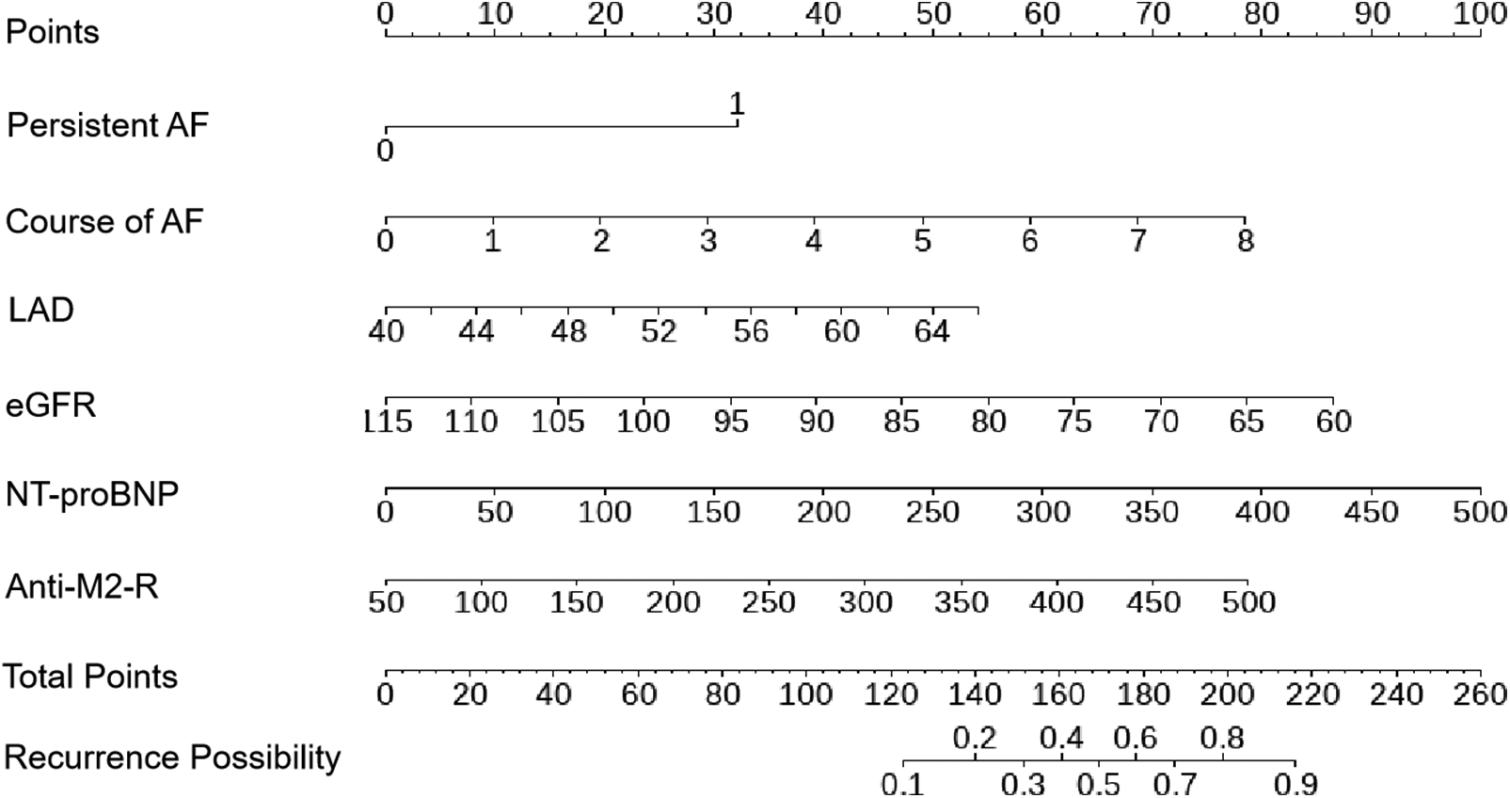
Nomogram model of AF recurrence after RFCA for AF patients.

A nomogram was constructed using the six variables, namely persistent AF, course of AF, eGFR, LAD, NT-proBNP, and anti-M2-R to predict AF recurrence after first-time RFCA during a 2-year follow-up period (Figure 3). The score for each factor can be obtained by locating the corresponding values on the vertical line. To calculate the total score of the nomogram, the scores of each factor were added. The corresponding prediction probability is the incidence of AF recurrence.

### 3.3 Model validation and net benefit prediction

**Figure 4:**
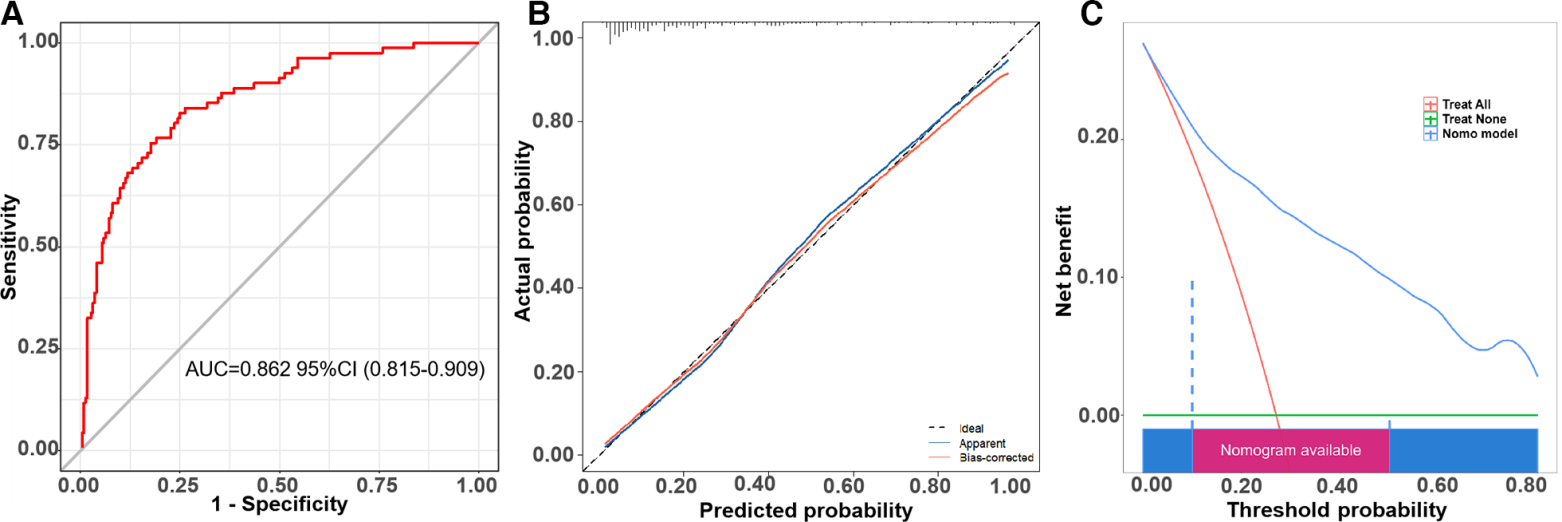
The ROC curve, calibration curve, and decision curve of the nomogram model for predicting AF recurrence in the training cohort. **(A)** ROC curve of the nomogram model for predicting AF recurrence. **(B)** Calibration curve of the nomogram model for predicting AF recurrence. **(C)** Decision curve of the nomogram model for predicting AF recurrence.

The receiver operator characteristic (ROC) curve for this new prediction model regarding the AF recurrence is shown in Figure 4A. The C-index was 0.862 [95% confidence interval (CI), 0.815–0.909] in the developing cohort. The calibration plot and DCA curves for this prediction model are shown in Figures 4B,C, respectively.

**Figure 5:**
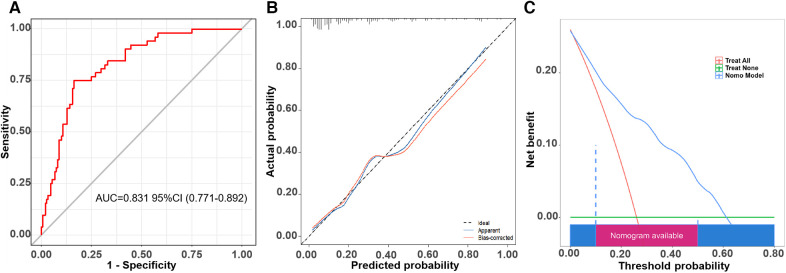
The ROC curve, calibration curve, and decision curve of the nomogram model for predicting AF recurrence in the validation cohort. **(A)** ROC curve of the nomogram model for predicting AF recurrence. **(B)** Calibration curve of the nomogram model for predicting AF recurrence. **(C)** Decision curve of the nomogram model for predicting AF recurrence.

We proved this prediction model in the validation cohort. The ROC curve is shown in Figure 5A, and the C-index was 0.831 (95% CI, 0.771–0.890). The calibration curve of this prediction model is shown in Figure 5B, suggesting a good coherence between the prediction results and the actual results for AF recurrence. In addition, the DCA showed that the nomogram had a high overall net benefit (Figure 5C).

### 3.4 Comparison with other models

**Figure 6:**
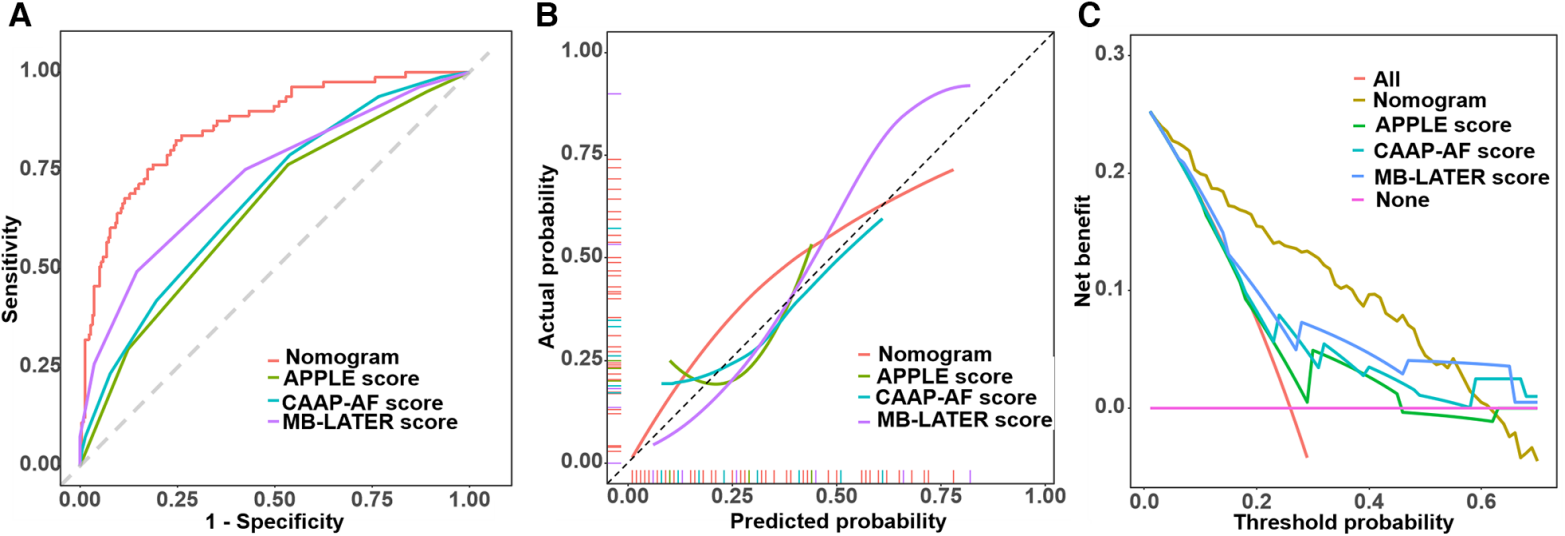
Comparison of ROC curves, calibration curves, and decision curves. **(A)** Comparison of ROC curves. **(B)** Comparison of calibration curves. **(C)** Comparison of decision curves.

To investigate the benefit of our new model, we calculated the APPLE score, CAAP-AF score, and MB-LATER score. The area under the curve (AUC) of the new model demonstrated significant differences compared with the APPLE score, CAAP-AF score, and MB-LATER score (Table 3, Figure 6A). We compared the nomogram with these models and evaluated the IDI and NRI indices. It can be seen that the IDI and NRI indices of the nomogram were noticeably higher than those of other models, indicating a positive improvement in efficacy and emphasizing its advantage in predicting the recurrence of AF for a 2-year period (Figure 6, Table 4).

**Table 3:** Comparison of prediction models.

Score	AUC	95% CI	*P* (compared with our new model)
Our new model	0.831	0.771–0.890	–
APPLE score	0.621	0.534–0.708	<0.001
CAAP-AF score	0.674	0.585–0.763	<0.001
MB-LATER score	0.698	0.617–0.779	<0.001

AUC, area under the curve; CI, confidence interval.

**Table 4:** Model validation and comparison between the nomogram and other models with NRI and IDI.

Our new model vs.	IDI	*P*-value	NRI	*P*-value
APPLE score	0.296	<0.001	0.361	<0.001
CAAP-AF score	0.243	<0.001	0.266	<0.001
MB-LATER score	0.196	<0.001	0.201	0.027

IDI, integrated discrimination index; NRI, net reclassification index.

## 4 Discussion

In this study, we developed a new prediction model for AF recurrence in patients who underwent first-time RFCA. The new prediction model based on six variables (persistent AF, course of AF, LAD, eGFR, NT-proBNP, and anti-M2-R) was associated with better predictive performance compared with the APPLE score, CAAP-AF score, and MB-LATER score for AF recurrence after RFCA during a 2-year follow-up period. The predicted efficacy of the new model was confirmed in an independent cohort. In conclusion, the results indicate that the nomogram demonstrated satisfactory discriminative power in both our training and validation cohorts of Chinese AF patients treated with first-time RFCA.

Lifestyle factors such as smoking, alcohol consumption, and endurance sports, as well as comorbidities such as hypertension, hyperthyroidism, and channelopathies, are associated with the development of AF (15–18). Considering that these factors may prevent future AF attacks regardless of the ablation strategy; we collected this information at baseline and excluded patients with hyperthyroidism, as we considered it to be an autoimmune disease. There were no statistical differences between the recurrence group and the non-recurrence group in terms of smoking, endurance sports, hypertension, or channelopathies. The LASSO regression analysis suggested that alcohol consumption was a factor influencing the recurrence of AF, but failed to be included in the multivariate model. Persistent AF, course of AF, LAD, eGFR, and NT-proBNP are common risk factors related to AF recurrence (19, 20). The correlation between the occurrence and development of AF and anti-M2-R has gradually been recognized. Anti-M2-R was first reported in patients with idiopathic dilated cardiomyopathy in 1993 (21). Subsequently, anti-M2-R was detected as an independent predictor for the concurrence of AF in patients with idiopathic dilated cardiomyopathy and Graves' hyperthyroidism (22). Anti-M2-R activates muscarinic-gated potassium channels by binding to the M2 receptor, leading to cell hyperpolarization and shortening of action potential duration. This results in significant fibrosis of atrial tissue in rabbits immunized with peptides corresponding to the second extracellular loop of M2 receptor (23). Serum levels of anti-M2-R may be associated with the degree of atrial fibrosis as quantified by delayed enhancement of MRI and Masson staining (12, 13). Some researchers have indicated that anti-M2-R may serve as a new mediator or upstream target for AF (24).

The nomogram showed superior clinical utility and higher accuracy than the APPLE score, the CAAP-AF score, and the MB-LATER score. The APPLE score, developed in early 2015, has the ability to predict AF recurrence in patients subjected to RFCA surgery. However, the CAAP-AF score did not show better clinical practicability or discrimination ability than this new prediction model. This difference may be due to variations in nationality, disease type and characteristics, medical techniques, and surgical methods.

The main advantages of our prediction model lie in its simplicity and utility. Constructed based on data from Chinese patients, our model is designed specifically to meet the needs of the population. Given the large number of AF patients and RFCA surgeries in China, our prediction model is expected to serve as a valuable tool for predicting AF recurrence in Chinese patients and assisting cardiologists in clinical practice.

### 4.1 Limitations

This single-center study underscores the necessity for external validation to ensure the clinical generalization and applicability of the model. In addition, our study was limited by its retrospective nature. Some biomarkers with potential predictive power could not be systematically monitored during the perioperative period and therefore were not included in this model. Consequently, further prospective multi-center studies are much needed.

## 5 Conclusion

We constructed and validated a new nomogram to predict the recurrence of AF with data from Chinese patients. This nomogram includes predictors such as persistent AF, duration of AF, LAD, eGFR, NT-proBNP, and anti-M2-R. Using a group of AF patients, this prediction model displayed better practicability and discrimination compared to the APPLE score, the CAAP-AF score, and the MB-LATER score. In conclusion, although further validation is needed, this prediction model may be an effective assistant for predicting the recurrence of AF in patients undergoing RFCA.

## Data Availability

The original contributions presented in the study are included in the article/Supplementary Material, further inquiries can be directed to the corresponding author.
